# Bias associated with the detectability of the coral-eating pest crown-of-thorns seastar and implications for reef management

**DOI:** 10.1098/rsos.170396

**Published:** 2017-08-02

**Authors:** Mohsen Kayal, Pauline Bosserelle, Mehdi Adjeroud

**Affiliations:** 1 Bren School of Environmental Science and Management, University of California, Santa Barbara, CA 93106-5131, USA; 2 EPHE, PSL Research University, UPVD, CNRS, USR 3278 CRIOBE and Laboratoire d'Excellence CORAIL, Papetoai, Moorea, French Polynesia; 3 Pacific community (SPC), Fisheries, Aquaculture and Marine Ecosystem division, BP D5, 98848 Noumea, New Caledonia; 4 Institut de Recherche pour le Développement, UMR 9220 ENTROPIE and Laboratoire d'Excellence CORAIL, Université de Perpignan, 52 avenue Paul Alduy 66860 Perpignan, France

**Keywords:** pest outbreak, *Acanthaster planci*, estimation bias, habitat complexity, density-dependence, contrast curve

## Abstract

Outbreaks of the predator crown-of-thorns seastar (COTS) *Acanthaster planci* cause widespread coral mortality across the Indo-Pacific. Like many marine invertebrates, COTS is a nocturnal species whose cryptic behaviour during the day can affect its detectability, particularly in structurally complex reef habitats that provide many refuges for benthic creatures. We performed extensive day and night surveys of COTS populations in coral reef habitats showing differing levels of structural complexity and COTS abundance. We tested whether estimations of COTS density varied between day and night observations, and if the differences were related to changes in COTS abundance, reef structural complexity and the spatial scale of observation. Estimations of COTS density were on average 27% higher at night than during the day. Differences in COTS detection varied with changing seastar abundance but not reef structural complexity or scale of observation. Underestimation of COTS abundance in daytime was significant for a broad seastar density range, thus potentially affecting most outbreak events. Our study suggests that portions of COTS populations can be undetected during conventional surveys and control campaigns, which are exclusively conducted by day, and significantly affect the trajectory of coral reefs. Accounting for bias in COTS detection can strengthen coral reef management broadly.

## 1. Introduction

Outbreaks of the voracious coral predator crown-of-thorns seastar *Acanthaster planci* (COTS) cause widespread coral decline and constitute major threats to reef health across the Indo-Pacific [1,2]. Increasing reef degradation and climate change are predicted to accentuate these events, further challenging reef conservation in the twenty-first century [3–6]. COTS outbreaks can result in drastic alteration of the physical and biological structure of coral reefs [7], with consequences for valuable resources they provide to human populations [8]. For example, 42% of coral decline and a multitude of socio-economic impacts are attributed to COTS outbreaks on the Australian Great Barrier Reef [9,10]. As a result, COTS is considered as a harmful species, a pest, in many regions [1,11,12]. Consequently, COTS densities are often surveyed as part of reef monitoring programmes, and population control campaigns are frequently conducted in an attempt to reduce coral decline [13–16], despite relatively high cost and limited efficiency [1,17]. However, like many marine invertebrates, COTS are predominantly nocturnal organisms that tend to hide in crevices and remain inactive during the day, while moving and feeding on corals by night, though this behaviour can be affected by many factors [18–22]. Despite the potential effect of such cryptic behaviour on COTS detectability during surveys and control campaigns, the efficiency of COTS detection by day versus night had not been thoroughly tested across spatio-temporal scales of observation and reef environments. Because monitoring and control operations are exclusively conducted during daytime, the nocturnal behaviour of COTS could be a major source of underestimation of COTS densities, thus affecting efficiency of ecological surveys, management decisions and control efforts on Indo-Pacific reefs.

We conducted paired day and night surveys of COTS densities around the island of Moorea, French Polynesia, during one of the most intense outbreaks ever documented [7]. Here, we test for differences in COTS abundance between day and night counts at different spatio-temporal scales of observation, and across reef habitats showing a wide range in structural complexity and COTS abundance.

## 2. Material and methods

We conducted our study during a particularly devastating COTS outbreak that affected the island of Moorea, French Polynesia [7]. Dense adult COTS aggregations, composed predominantly of 30–45 cm diameter individuals, emerged on the north shore of the island in 2003 and gradually expanded to the entire insular reef system. By 2006, all reef habitats (fringing, barrier, outer-slope) and the three coasts of the island (north, east, west) were affected, constituting a widespread coral-mortality event. The outbreak ended in 2010, leaving reefs largely denuded of coral cover and with altered benthic and fish communities [7]. Outbreaks similar in dynamics and impacts were simultaneously observed on other proximal islands of French Polynesia. At a broader scale, these outbreaks seem to be part of a larger cycle of regional outbreaks that expanded across the Indo-Pacific, from Polynesia to the Red Sea [23].

**Figure 1.**
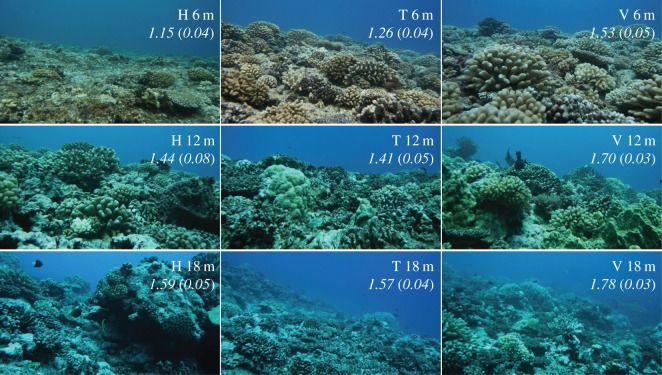
Variability in reefscape across sites and depths as observed around Moorea, French Polynesia. The nine reef locations consisted of three water depths (vertically; 6 m, 12 m, 18 m) at each of three sites (horizontally; H, Haapiti; T, Tiahura; V, Vaipahu). Values in italic indicate mean (s.e.) substrate rugosity as estimated in 2008 by the chain-and-tape method. Note that reef structural complexity varies in time and space with changing coral community abundance and structure. Pictures were taken in November 2007.

COTS densities around Moorea were surveyed throughout the outbreak at multiple scales, including semestrial counts that were performed in triplicate 200 m^2^ (50 m × 4 m) permanent-transects at nine outer-reef locations consisting of three water depths (6 m, 12 m, 18 m) at each of three sites (Haapiti, Tiahura, Vaipahu) [24]. These reef habitats showed contrasting structural complexity (figure 1) and COTS abundances [7], thus providing a good test of COTS detectability across a broad range in physical habitat structure and intensity of outbreaks. Indeed, average COTS densities over the process of this study varied between nil (0 seastar 200 m^−2^) and one of the highest levels reported in the literature (30.3 seastar 200 m^−2^, equivalent to 151 650 seastar km^−2^). This range expands far beyond the estimated maximum sustainable density for coral communities of 1000–1500 seastar km^−2^ [20,25]. Between October 2007 and October 2009, we paired each of our diurnal surveys of COTS densities at the nine reef locations with a nocturnal survey performed within 12 h by the same group of divers equipped with flashlights in the same permanent-transects [24]. Diurnal surveys were performed between 09.00 and 15.00, and nocturnal surveys between 21.00 and 00.00, allowing for at least 3 h before and after sunrise and sunset. All surveys were performed on SCUBA.

We tested for differences in COTS densities between day and night counts using generalized linear mixed-effect models [26] that accounted for repeated, hierarchically designed observations performed at different dates in individually distinct transects established at specific sites and depths. Variability in response variable *density* was tested against the explicative factor *timing-of-observation* with random effects of the variables *date*, *transect*, *depth* and *site*. We tested if differences in density between day and night counts varied with the spatio-temporal scale considered by running separate models with explicative interaction terms *timing-of-observation* × each specific scale of observation, from individual transects (covering 200 m^2^ of reef area) to reef locations (approx. 1000 m^2^), sites (approx. 5000 m^2^ along continuous reef slope) and depths (approx. 5000 m^2^ along the reef isodepth across 20 km of coastline), and within single dates of observation versus over the entire process of the study (2 years).

We also tested whether differences in COTS density between day and night counts were correlated with changes in reef structural complexity, COTS abundance and their interaction. Reef structural complexity was quantified in 2008 using the chain-and-tape method [27] over a 10 m linear portion of each of the permanent-transects used to perform the COTS surveys [24]. A chain following the contour of the substrate was laid over the reef surface, and rugosity was calculated as the ratio between the contoured chain-length and the 10 m linear distance. As differences in COTS density correlated with COTS abundance, we used the semi-parametric contrast curve approach as a *post hoc* test to identify the range in seastar abundance for which estimations of COTS density differed significantly between day and night counts [28,29]. Contrast curves combine generalized linear mixed-effect model and penalized-spline statistics for an optimized representation of variability in data, including within-subject differences (in this case, observations that were replicated in time and space) and nonlinear responses [30]. Methodology and programing code for the contrast curve approach are provided in previous studies [28,29]. COTS abundance was log(*x *+ 1) transformed before data analysis to homogenize residual variance. All statistics and graphing were coded in R (R Development Core Team) complemented by the NLME package [26].

## 3. Results

**Figure 2.**
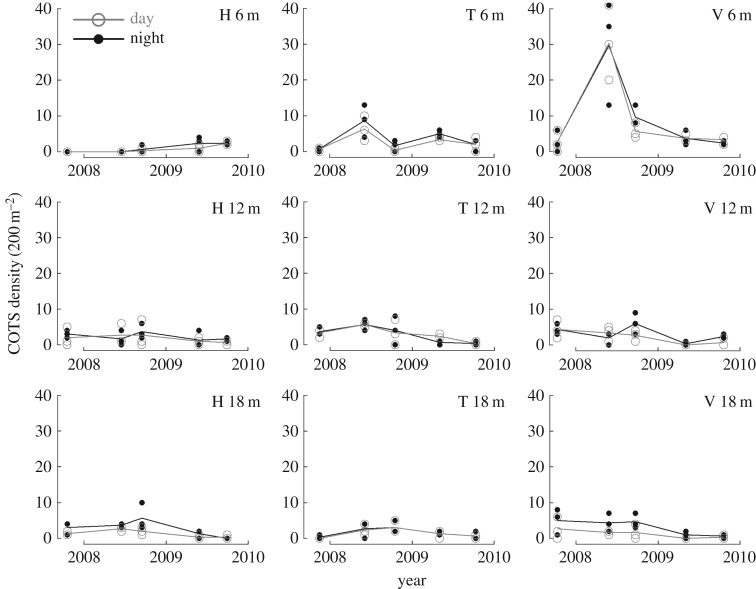
Density trajectories of the coral-killing seastar COTS as counted by day and night time. Surveys were performed in permanent-transects established at nine reef locations that consisted of three water depths (vertically; 6 m, 12 m, 18 m) at each of three sites (horizontally; H, Haapiti; T, Tiahura; V, Vaipahu). Dots represent actual observations and lines illustrate mean trajectories.

COTS densities followed similar trajectories between day and night observations (figure 2). However, significantly higher density-values were recorded at night over the process of the study and across all reef locations (*p *< 0.001). Overall, estimated COTS densities were on average 27% higher at night (mean = 3.3 (standard error = 0.4 s.e.) seastar 200 m^−2^, equivalent to 16 500 seastar km^−2^) than during the day (2.6 (0.4 s.e.) seastar 200 m^−2^, equivalent to 13 000 seastar km^−2^).

**Figure 3.**
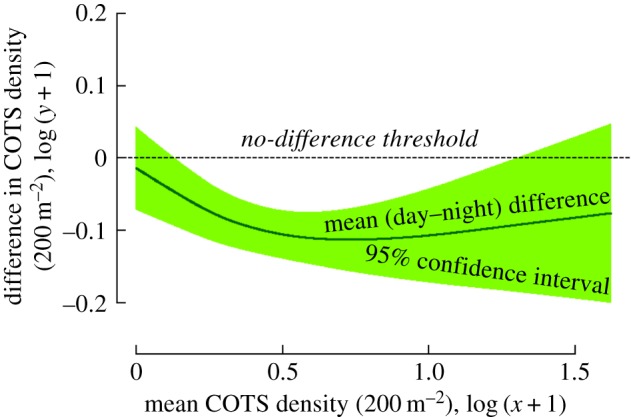
Contrast curve identifying the domain of significant difference in estimations of seastar density between day and night surveys. Densities of the coral-killing seastar COTS were estimated every six months over a period of 2 years by day and night counts performed in permanent-transects established at nine reef locations (figure 2). The semi-parametric contrast curve [28,29] represents variation in the difference between day and night estimations (difference = day density − night density, *y*-axis) along the seastar density range (*x*-axis). The domain of significant difference is identified as the portion of the covariate (*x*-axis) for which the 95% confidence interval of the contrast curve (shaded area) does not cross the no-difference threshold (horizontal dashed line): COTS abundance was significantly lower in day counts compared to night in the log-density range 0.13–1.31, corresponding to the COTS density interval 0.4–19.3 seastar 200 m^−2^.

Differences in COTS density between day and night observations were not significantly variable between different dates of observation (*p *= 0.078) and across spatial scales, from individual transects (*p *= 0.598), to reef locations (*p *= 0.067), sites (*p *= 0.154) and depths (*p *= 0.114). Differences between day and night counts were not correlated to changes in reef habitat complexity as measured by the rugosity index, neither in isolation (*p *= 0.239) nor in interaction with COTS abundance (*p *= 0.733). By contrast, differences between day and night counts varied with COTS density (*p *= 0.009). Based on the semi-parametric contrast curve approach (figure 3), COTS densities were significantly lower during day counts compared with night time over the density range 0.4–19.3 seastar 200 m^−2^ (equivalent to 2000–96 500 seastar km^−2^).

## 4. Discussion

Our evaluation of COTS surveys in Moorea indicated a 27% underestimation of population densities during daytime compared to nocturnal observations. These differences in estimations of COTS density were consistent in time, space and across multiple scales of observation, but varied with changing COTS abundance. Across the wide range in reef habitat structure and COTS abundance that the reefs provided, we estimated that day counts significantly underestimated COTS density compared with night counts within the seastar abundance range of 2000–96 500 km^−2^. Underestimation of COTS density might be commonplace because surveys and control campaigns are exclusively conducted by day, and most reports of COTS abundance during outbreaks fall within this range [1].

Our results are in concordance with a previous investigation that found higher abundances of COTS by night due to a more cryptic behaviour of smaller (less than 20 cm diameter) seastars in daylight [21]. By contrast, a lower detection of tagged COTS individuals at night compared to daytime was reported by a recent study [11]. Discrepancy in COTS detectability at day versus night can result from different mechanisms that act in antagonistic ways, given the cryptic behaviour of COTS during daytime on one side, and the limited human sight at night on the other. In particular, these mechanisms are further influenced by numerous additional factors such as the dedicated search effort and experience of observer divers, weather conditions, various characteristics of reef habitats such as water depths and levels of structural complexity, and the abundance, size and movement of the seastars in and out of the search zone ([1,11,13,18,21,22,31], this study). The sizes of individual seastars were not measured during our surveys although, as in the recent study [11], COTS populations were composed predominantly of large (greater than 30 cm diameter) individuals. Other methodological differences included smaller sample sizes and tagging of the seastars in the recent study, whereas we performed non-invasive observations that were spatio-temporally replicated within permanent-transects without discriminating individual seastars. Besides, COTS densities were lower during the recent study [11], and surveys were performed in fragmented habitats compared with our investigation of a dynamic outbreak evolving through continuous reefs that showed a wide range in structural complexity, COTS abundance and availability in prey corals [7].

COTS outbreaks have been documented since the 1950s, but a new cycle of regional outbreaks throughout the Indo-Pacific has sparked heightened interest [1,23]. The amplitude of coral decline associated with these recent events is astounding, as might be the consequential loss in coral reef resources and ecological services [8,9]. Nevertheless, the recent outbreaks provided unique opportunities to further understanding of COTS biology and ecology, including developing strategies for detecting and controlling future outbreaks [12,14,17,32,33] and identifying ecological processes that can promote coral reef resilience in the face of these disturbances [23,34–36]. However, potential bias in COTS survey methodology might preclude efficient management of coral reef resources. Our study suggests that underestimation of COTS abundance might be commonplace, potentially resulting in under-recognition of COTS outbreaks as well as unsuccessful control efforts. Indeed, an adult COTS is estimated to consume 160–480 cm^2^ of coral tissue every day, and a particularly high fecundity promotes the resurgence of widespread COTS populations even from few seastars [1,20,37]. A 27% difference in COTS abundance, as estimated in our study, can thus drastically change coral reef trajectory.

COTS are expected to be increasingly more prominent protagonists of coral reefs in the near future. We advocate accounting for detection bias in conventional surveys and control campaigns, or complementing them with observations performed by night, in order to strengthen detection and control of outbreaks. Alternatively, COTS surveys can be complemented by indirect measurement protocols such as DNA-sequencing reef water samples [12,33] or monitoring the characteristic feeding scars that the seastar leaves on corals that are preyed upon [7,20,38].

## Data accessibility

Crown-of-thorns seastar (COTS) density and reef substrate rugosity data used in this study is available from the Dryad Digital Repository: http://dx.doi.org/10.5061/dryad.dk5dm [24].
